# Both low and high serum ferritin levels predict mortality risk in hemodialysis patients without inflammation

**DOI:** 10.1007/s10157-016-1317-1

**Published:** 2016-08-08

**Authors:** Tetsuo Shoji, Kakuya Niihata, Shingo Fukuma, Shunichi Fukuhara, Tadao Akizawa, Masaaki Inaba

**Affiliations:** 1 Department of Vascular Medicine, Osaka City University Graduate School of Medicine, 1-4-3, Asahi-machi, Abeno-ku, Osaka, 545-8585 Japan; 2 Institute for Health Outcomes and Process Evaluation research (iHope International), Kyoto, Japan; 3 Center for Innovative Research for Communities and Clinical Excellence (CIRC2LE), Fukushima Medical University, Fukushima, Japan; 4 Department of Healthcare Epidemiology, School of Public Health in the Graduate School of Medicine, Kyoto University, Kyoto, Japan; 5 Division of Nephrology, Department of Medicine, Showa University School of Medicine, Tokyo, Japan; 6 Department of Metabolism, Endocrinology and Molecular Medicine, Osaka City University Graduate School of Medicine, Osaka, Japan

**Keywords:** Ferritin, Mortality, Cardiovascular disease (CVD), Hemodialysis, Cohort

## Abstract

**Background:**

Serum ferritin concentration >100 ng/mL was associated with a higher risk of death in hemodialysis patients in Japan, whereas such an association was less clear in hemodialysis patients in Western countries. Since Japanese dialysis patients are generally less inflamed than those in Western countries, inflammation may modify the association between serum ferritin and the adverse outcomes.

**Methods:**

We performed an observational cohort study using data from 2606 Japanese hemodialysis patients who participated in the Dialysis Outcomes and Practice Patterns Study (DOPPS) III (2005–2008) or DOPPS IV (2009–2012). The predictor was serum ferritin category (<50, 50–99.9, 100–199.9, and ≥200 ng/mL), and the primary and secondary outcomes were all-cause mortality and cardiovascular hospitalization, respectively. C-reactive protein (CRP, cut-off by 0.3 mg/dL) and serum albumin (cut-off by 3.8 g/dL) were stratification factors related to systemic inflammation.

**Results:**

After adjustment for relevant confounding factors, a U-shaped association was observed between serum ferritin and all-cause mortality in the group with low CRP levels, whereas such relationship was not significant in the high CRP counterparts. In contrast, we found a linear association between serum ferritin and cardiovascular hospitalization in the low CRP and high CRP groups commonly. Similar results were obtained when the total cohort was stratified by serum albumin.

**Conclusions:**

Serum ferritin showed different patterns of association with all-cause mortality in hemodialysis patients with versus without inflammation, whereas its association with cardiovascular hospitalization was similar regardless of inflammatory conditions.

## Introduction

Serum ferritin concentration is clinically used as a marker of iron store, and it is also an independent predictor of mortality and other adverse outcomes in hemodialysis patients. Kalantar-Zadeh et al. [1] previously showed that serum ferritin correlated positively with hospitalization days and frequency, and that the increase in it predicted death in 12 months in 101 hemodialysis patients. They performed another historical cohort study in 58,058 maintenance hemodialysis [2] showing a higher risk for all-cause and cardiovascular mortality only in serum ferritin ranges >1200 ng/mL as compared with the reference range (100–199 ng/mL) in models adjusted for case-mix variables. When further adjustment for 9 variables for the malnutrition–inflammation–cachexia syndrome, the association between high serum ferritin and mortality was much less significant. Recently, Karaboyas et al. [3] reported a trend of increase in serum ferritin in the US after the implementation of the new bundled payment system in the US, with the median level of serum ferritin of 601 ng/mL in 2009 and 887 ng/mL in 2012.

In contrast to the above studies in the US, Hasuike et al. [4] found in a cohort of 90 Japanese hemodialysis patients that serum ferritin >100 ng/mL was associated with higher mortality risk than those with lower ferritin levels. Recently, Kuragano et al. [5] performed a prospective cohort study of 1086 Japanese maintenance hemodialysis patients with repeated measurements of serum ferritin, showing that the risk of death and/or adverse events was higher in those whose ferritin levels were kept high and in those showing large fluctuation in ferritin. In their study, high ferritin was defined as >100 ng/mL based on the mean and median serum ferritin levels (127 and 79 ng/mL, respectively) measured during the study period. Thus, there are considerable international differences in serum ferritin concentrations and in the association between serum ferritin concentration and adverse outcomes, which may affect the upper limit of serum ferritin concentration in the management of anemia in hemodialysis patients. However, the reason is unknown why such difference exists in the ferritin–mortality relationship among countries.

Based on the facts that serum ferritin is lower in Japanese hemodialysis patients than those in other countries [6], that serum ferritin is increased in the presence of inflammation [7], and that the Japanese dialysis patients are generally less inflamed as shown by a lower C-reactive protein (CRP) level than those in the Western countries [8, 9], we hypothesized that the association between serum ferritin and the adverse outcomes could be modified by the presence of inflammation.

## Methods

### Study population

The Dialysis Outcomes and Practice Patterns Study (DOPPS) is a prospective cohort study of outcomes and practice patterns for hemodialysis patients. The method used for sampling the study participants has been described in detail previously [10, 11]. In the present study, data from the Japanese DOPPS (J-DOPPS) III and IV studies were used. J-DOPPS III (*n* = 2556) and J-DOPPS IV (*n* = 2551) were conducted between 2005 and 2008 and between 2009 and 2012, respectively. Patients who had a high CRP level exceeding 50 mg/dL were excluded because they may have been clinically unstable due to severe infection.

### Exposure and outcomes

The exposure variable was the serum ferritin level measured at the baseline of J-DOPPS III or IV. It was categorized into 4 groups according to the following cut-off values: 50, 100, and 200 ng/mL. The cut-off values of 100 and 200 ng/mL are those for iron therapy supplementation, based on the 2008 guidelines of the Japanese Society for Dialysis Therapy (JSDT) [12] and the National Kidney Foundation Kidney Disease Outcomes Quality Initiative (NKF KDOQI) [13], respectively. An additional cut-off level of 50 ng/mL was set as a half-value of 100 ng/mL, because there is no well-established cut-off value for absolute iron deficiency.

The primary outcome was all-cause mortality, and the secondary outcome was hospital admission due to cardiovascular disease (CVD) including congestive heart failure, coronary artery disease, peripheral artery disease, cerebrovascular disease and others (pericarditis, cardiac arrest, chronic atrial fibrillation, other arrhythmias, implantation of permanent pacemaker or automatic implanted cardiac defibrillator, valvular heart disease, and prosthetic heart valve replacement). The same definition was used for the pre-existing CVD at baseline.

Patients were censored at the time of kidney transplantation, transfer to another dialysis unit, or the end of the observation period of J-DOPPS III or IV.

### Statistical analysis

Baseline characteristics were presented in terms of standard descriptive statistics: mean (standard deviation) and median (25th percentile and 75th percentile) for the 4 groups. The Cox proportional hazards model was used to estimate the hazard ratios (HRs) and 95 % confidence intervals (95 %CI) for outcome in terms of the exposure variable using univariate and multivariate analysis. In multivariate analysis, the model was adjusted according to relevant covariates: age, gender, years on dialysis, the presence of diabetes mellitus, history of CVD, body mass index, the levels of serum albumin, serum C-reactive protein (CRP), hemoglobin, single pool Kt/V, dose of ESA, and dose of IV iron at baseline.

Stratified analyses were performed to evaluate the interaction of inflammation on the association between serum ferritin and outcomes. The total cohort was stratified either by serum CRP level (cut-off 0.3 mg/dL) or by serum albumin level (cut-off 3.8 g/dL), and the same analyses were applied to each stratum of patients. The cut-off value for CRP of 0.3 mg/dL was based on a previous J-DOPPS study by Kawaguchi et al. [8] reporting the association between CRP and mortality. Because there is no established cut-off level for serum albumin as a marker of inflammation, we used a serum albumin level of <3.8 g/dL, which has been proposed by Fouque et al. [14] as a readily applicable criterion for clinical diagnosis of protein-energy wasting (PEW) in patients with acute and chronic kidney disease. A *P* value of <0.05 was considered to indicate statistical significance. All analyses were performed without any imputation methods using STATA version 13 (Stata Corp LP, College Station, TX, USA).

### Ethical considerations

This study was performed in accordance with the Declaration of Helsinki. The protocols of J-DOOPS III and J-DOPPS-IV were reviewed and approved by the Ethics Committee of Tokyo Women’s Medical University (Approval Numbers 709, 1178, 1278, 1527, 1826, and 2143). All participants gave informed consent before enrollment.

## Results

### Baseline characteristics of the cohort

**Fig. 1:**
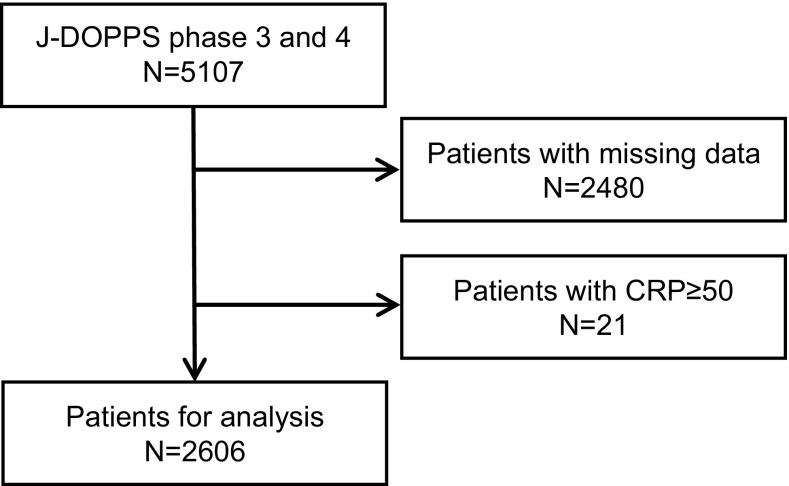
Selection of the subjects

**Fig. 2:**
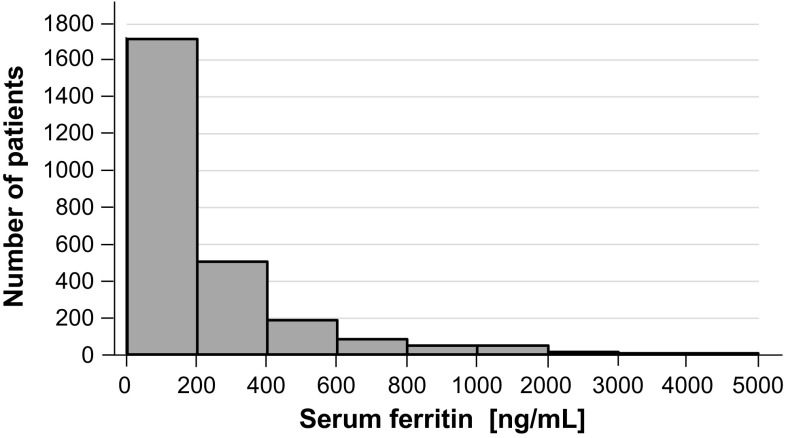
Distribution of serum ferritin concentrations

A total of 5107 patients were enrolled in J-DOPPS III and IV. After excluding 2480 patients because of missing data and 21 because of CRP level of ≥50 mg/dL, 2606 patients were enrolled for the final analyses (Fig. 1). The distribution of serum ferritin levels among the study population is shown in Fig. 2. The Kidney Disease Improving Global Outcomes (KDIGO) guideline published in 2012[15] recommends intravenous (IV) iron supplementation in those with serum ferritin <500 ng/mL. The proportion of patients with ferritin levels of >500 ng/mL was low in J-DOPPS (11.4 %). The baseline characteristics of the patients overall and in the 4 groups by serum ferritin level are summarized in Table 1. The mean (SD) patient age was 64.0 (12.0) years and the median (interquartile range) years on dialysis were 5.0 (1.63–10.3) years. In terms of laboratory data, the mean (SD) serum albumin level was 3.76 (0.42) g/dL and the median (interquartile range) serum CRP level was 0.12 (0.06–0.37) mg/dL.

**Table 1:** Baseline characteristics of the cohort

Variable	Total cohort (*n* = 2606)	Serum ferritin category			
		<50 ng/mL (*n* = 616)	50–99.9 ng/mL (*n* = 450)	100–199.9 ng/mL (*n* = 635)	≥200 ng/mL (*n* = 905)
Age (years)	64.0 (12.0)	62.9 (12.0)	62.7 (12.4)	64.4 (11.9)	65.0 (11.7)
Male (%)	62.1	63.0	60.2	58.9	64.6
Years on dialysis (years)	5.0 (1.6–10.3)	5.8 (2.2–10.7)	4.9 (1.30–10.4)	4.7 (1.2–10.4)	4.9 (1.5–10.1)
Diabetes (%)	38.7	33.0	39.3	38.0	42.9
Comorbidities of CVD (%)					
CHF	25.7	25.7	25.6	23.9	27.0
CAD	37.5	37.0	37.3	35.0	39.6
Stroke	15.2	14.1	18.4	13.1	15.9
PAD	20.2	17.2	20.0	19.7	22.8
Others	33.7	30.4	33.1	32.1	37.4
Body mass index (kg/m^2^)	21.1 (3.3)	21.3 (3.3)	21.1 (3.4)	21.1 (3.3)	21.1 (3.3)
Alb (g/dL)	3.76 (0.42)	3.81 (0.41)	3.76 (0.41)	3.76 (0.40)	3.74 (0.43)
CRP (mg/dL)	0.12 (0.06–0.37)	0.12 (0.06–0.31)	0.10 (0.05–0.30)	0.10 (0.06–0.31)	0.15 (0.06–0.50)
Hb (g/dL)	10.4 (1.2)	10.4 (1.3)	10.5 (1.2)	10.4 (1.2)	10.3 (1.2)
Single pool Kt/V	1.35 (0.28)	1.38 (0.27)	1.34 (0.31)	1.34 (0.28)	1.34 (0.28)
ESA users (%)	92.0	90.0	93.2	92.0	92.7
Dose of ESA (10^3^ units/month)	18.0 (9.0–28.5)	18.0 (11.3–36.0)	16.5 (9.8–26.4)	18.0 (9.0–24.0)	16.0 (9.0–27.0)
IV iron user (%)	30.0	24.7	32.0	30.5	32.1
Dose of IV iron (mg/month)	160 (160–320)	160 (160–320)	160 (160–480)	160 (160–480)	160 (160–240)

Values for categorical variables are given as percentage; values for continuous variables are given as mean (SD) or median (interquartile range). Dose of ESA was calculated only for ESA users and expressed as recombinant human erythropoietin (epoetin α or ß) using a conversion ratio of 1:200 for darbepoetin α. Dose of IV iron was also calculated only for IV iron users

*Alb* albumin, *Hb* hemoglobin, *CRP* C-reactive protein, *CVD* cardiovascular disease, *CHF* congestive heart failure, *CAD* coronary artery disease, *PAD* peripheral artery disease, *ESA* erythropoiesis-stimulating agent, *IV* intravenous

### Primary outcome

During a median follow-up period of 2.66 years, there were 283 deaths. The proportion of deaths during the follow-up period according to the ferritin categories is shown in Table 2. The proportion in the group whose ferritin levels were between 50 and 99.9 ng/mL was 7.3 %, representing the lowest proportion among the 4 groups. We estimated the HRs for all-cause mortality in comparison with the reference category (ferritin 50–99.9 ng/mL). The HRs in the univariate and multivariate analyses in the total cohort are shown in Table 3. Multivariate analysis showed that the group with the lowest ferritin level (HR, 1.79; 95 % CI 1.18–2.72) and that with the highest ferritin level (HR, 1.55; 95 % CI 1.05–2.29) were each significantly associated with all-cause mortality.

**Table 2:** Proportions of deaths and cardiovascular hospitalizations during follow-up according to the ferritin categories

Strata of subjects	Outcomes	Serum ferritin category				
		<50 ng/mL	50–99.9 ng/mL	100–199.9 ng/mL	≥200 ng/mL	Total
Total cohort	Number of subjects	616	450	635	905	2606
	Death	72 (11.7 %)	33 (7.3 %)	68 (10.7 %)	110 (12.2 %)	283 (10.9 %)
	CVD hospitalization	118 (19.2 %)	133 (29.6 %)	217 (34.2 %)	360 (39.8 %)	828 (31.8 %)
CRP <0.3 mg/dL	Number of subjects	440	328	454	583	1805
	Death	42 (9.5 %)	15 (4.6 %)	36 (7.9 %)	55 (9.4 %)	148 (8.2 %)
	CVD hospitalization	85 (19.3 %)	98 (29.9 %)	153 (33.7 %)	249 (42.7 %)	585 (32.4 %)
CRP ≥0.3 mg/L	Number of subjects	176	122	181	322	801
	Death	30 (17.0 %)	18 (14.8 %)	32 (17.7 %)	55 (17.1 %)	135 (16.9 %)
	CVD hospitalization	33 (18.8 %)	35 (28.7 %)	64 (35.4 %)	111 (34.5 %)	243 (30.3 %)
Alb ≥3.8 g/dL	Number of subjects	290	188	263	388	1129
	Death	20 (6.9 %)	4 (2.1 %)	21 (8.0 %)	40 (10.3 %)	85 (7.5 %)
	CVD hospitalization	55 (19.0 %)	58 (30.9 %)	89 (33.8 %)	165 (42.5 %)	367 (32.5 %)
Alb <3.8 g/dL	Number of subjects	326	262	372	517	1477
	Death	52 (16.0 %)	29 (11.1 %)	47 (12.6 %)	70 (13.5 %)	198 (13.4 %)
	CVD hospitalization	63 (19.3 %)	75 (28.6 %)	128 (34.4 %)	195 (37.7 %)	461 (31.2 %)

The table gives the numbers of subjects at baseline, and the numbers (percentages) of deaths and CVD hospitalizations during the follow-up in each category of serum ferritin in the total cohort as well as in the strata by CRP or serum albumin

*Alb* albumin, *CRP* C-reactive protein, *CVD* cardiovascular disease

**Table 3:** Associations of serum ferritin categories with all-cause mortality and CVD hospitalization for the total cohort

Outcomes	Ferritin categories (ng/mL)	Univariate analysis		Multivariate analysis^a^	
		HR	95 % CI	HR	95 % CI
All-cause mortality					
	<50	1.58	1.05–2.38	1.79	1.18–2.72
	50–99.9	1.00	Referent	1.00	Referent
	100–199.9	1.46	0.96–2.20	1.43	0.94–2.18
	≥200	1.70	1.15–2.51	1.55	1.05–2.29
CVD hospitalization					
	<50	1.00	Referent	1.00	Referent
	50–99.9	1.47	1.15–1.89	1.43	1.11–1.83
	100–199.9	1.55	1.24–1.94	1.53	1.22–1.92
	≥200	1.82	1.47–2.24	1.72	1.39–2.12

*CRP* C-reactive protein, *CVD* cardiovascular disease, *HR* hazard ratio, *ESA* erythropoiesis-stimulating agent, *IV* intravenous, *95* *% CI* 95 % confidence interval

^a^Adjusted for age, sex, years on dialysis, diabetes mellitus, prior CVD, body mass index, albumin, CRP, hemoglobin, and single pool Kt/V, ESA dose, and IV iron dose

**Fig. 3:**
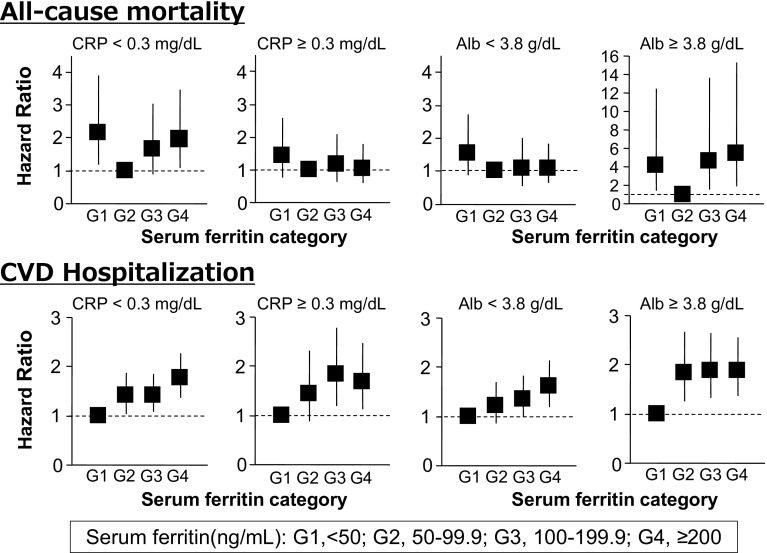
Associations of serum ferritin category with all-cause mortality and CVD hospitalization in strata by CRP and serum albumin. The *graphs* show hazard ratios (*squares*) and 95 % confidence intervals (*vertical* *lines*) in Cox models adjusted for age, sex, years on dialysis, diabetes mellitus, prior CVD, body mass index, serum albumin, CRP, single pool Kt/V, dose of ESA, and dose of IV iron. *CRP* C-reactive protein, *Alb* serum albumin, *CVD* cardiovascular disease, *G1 to G4*, categories of serum ferritin, *ESA* erythropoiesis-stimulating agent, *IV* intravenous

The HRs stratified by serum CRP levels are shown in Fig. 3. In the stratum with low CRP levels, the group with the lowest ferritin level (HR, 2.15; 95 % CI 1.18–3.90) and that with the highest ferritin level (HR, 1.95; 95 % CI 1.10–3.48) were each significantly associated with all-cause mortality by multivariate analysis. On the other hand, in the stratum with high CRP levels, none of the ferritin level groups had any significant association with all-cause mortality.

The HRs stratified by serum albumin levels are shown in Fig. 3. In the stratum with high serum albumin levels, patients with the lowest ferritin level had a significant association with mortality (HR, 4.20; 95 % CI 1.42–12.42). The two groups with high ferritin levels also showed a significant association with all-cause mortality (HR, 4.60; 95 % CI 1.56–13.63 in the 100–199.9 ng/mL group: HR, 5.41; 95 % CI 1.92–15.28 in the ≥200 ng/mL group). On the other hand, in the stratum with low serum albumin levels, none of the ferritin level groups showed any significant association with all-cause mortality.

Table 4 shows the causes of death in the four categories of serum ferritin. As compared with the reference ferritin category (50–99.9 ng/mL), the proportion of death due to gastrointestinal bleeding was numerically higher in the lowest ferritin category, whereas the proportion of cardiovascular death was similar between the two ferritin categories. Such trend was also found in the lower CRP stratum, but not in the higher CRP stratum.

**Table 4:** Cause of death according to the ferritin categories stratified by CRP level

	Serum ferritin category (ng/mL)			
	<50	50–99.9	100–199.9	≥200
Total cohort				
Number of subjects	616	450	635	905
Number of death				
Total	72 (100)	33 (100)	68 (100)	110 (100)
Cardiovascular disease	22 (30.6)	10 (30.3)	21 (30.9)	26 (23.6)
Infection	10 (13.9)	8 (24.4)	7 (10.3)	21 (19.1)
Malignancy	8 (11.1)	6 (18.2)	4 (5.9)	12 (10.9)
Gastrointestinal bleeding	3 (4.2)	0 (0)	1 (1.5)	0 (0)
Others	9 (12.5)	1 (3.0)	11 (16.2)	12 (10.9)
Unknown	20 (27.8)	8 (24.2)	24 (35.3)	39 (35.5)
CRP < 0.3 mg/dL				
Number of subjects	440	328	454	583
Number of death				
Total	42 (100)	15 (100)	36 (100)	55 (100)
Cardiovascular disease	14 (33.3)	5 (33.3)	13 (36.1)	10 (18.2)
Infection	4 (9.5)	3 (20.0)	2 (5.6)	9 (16.4)
Malignancy	5 (11.9)	4 (26.7)	2 (5.6)	6 (10.9)
Gastrointestinal bleeding	2 (4.8)	0 (0)	1 (2.8)	0 (0)
Others	5 (11.9)	0 (0)	5 (13.9)	6 (10.9)
Unknown	12 (28.6)	3 (20.0)	13 (36.1)	24 (43.6)
CRP ≥ 0.3 mg/dL				
Number of subjects	176	122	181	322
Number of death				
Total	30 (100)	18 (100)	32 (100)	55 (100)
Cardiovascular disease	8 (26.7)	5 (27.8)	8 (25.0)	16 (29.1)
Infection	6 (20.0)	5 (27.8)	5 (15.6)	12 (21.8)
Malignancy	3 (10.0)	2 (11.1)	2 (6.3)	6 (10.9)
Gastrointestinal bleeding	1 (3.3)	0 (0)	0 (0)	0 (0)
Others	4 (13.3)	1 (5.6)	6 (18.8)	6 (10.9)
Unknown	8 (26.7)	5 (27.8)	11 (34.4)	15 (27.3)

The table gives the numbers of subjects, the numbers (percentages) of death in each category of serum ferritin

### Secondary outcome

During a median follow-up period of 2.59 years, there were 828 CVD hospitalizations. The proportion of CVD hospitalizations during the follow-up period according to the ferritin categories are shown in Table 2. The proportion of hospitalizations in the group with the lowest ferritin level was 19.2 %, being the lowest among the 4 groups. We estimated the HRs for CVD hospitalization with serum ferritin categories with 50–99.9 ng/mL as the reference group. The HRs in univariate and multivariate analyses are shown in Table 3. The higher ferritin categories had higher HRs for CVD hospitalization. The association between serum ferritin and CVD hospitalization was not altered by stratification by CRP or serum albumin levels (Fig. 3).

## Discussion

In this study, we addressed a question whether the associations between serum ferritin and adverse clinical outcomes are modified by the presence of inflammation in hemodialysis patients using the dataset of J-DOPPS III and IV. In the stratum with low CRP levels, serum ferritin showed a U-shaped association with all-cause mortality after adjustment for relevant confounding factors. However, such relationship was not significant in the high CRP counterparts. Similar results were obtained when the total cohort was stratified by serum albumin. In contrast, we found a linear and positive association between serum ferritin and CVD hospitalization regardless of the levels of the serum markers of inflammation. These results support the view that the association between serum ferritin and all-cause mortality is modified by inflammation, whereas the presence of inflammation does not apparently modify the relationship between serum ferritin and CVD hospitalization.

We report here that the association between serum ferritin and mortality risk was different in the presence and the absence of inflammation. Kalantar-Zadeh et al. [2] were the first that reported the inter-relationship among serum ferritin, inflammation, and mortality risk of hemodialysis patients. They showed in the US hemodialysis patients that mortality risk was higher only in serum ferritin ranges >1200 ng/mL as compared with the reference range between 100 and 199 ng/mL, but that the hazard ratios were close to 1 (no association) in models further adjusted for variables related to malnutrition and inflammation. The mean serum ferritin concentration was 556 ng/mL in those who received IV iron at baseline and 718 ng/mL in those who did not receive it. They discussed that the increased mortality risk in those with high serum ferritin concentration was due mostly to the confounding effects by systemic inflammation and/or malnutrition. In contrast, a recent study by Park et al. [7] showed that the groups with high serum ferritin level (>142.6 ng/mL) showed a higher all-cause mortality risk than those with lower serum ferritin levels, and that the association between serum ferritin and mortality risk was independent of systemic inflammation and nutritional status in incident hemodialysis patients by including CRP as one of the covariates in the Cox model. The median serum ferritin in the Korean hemodialysis cohort was 213 ng/mL, much lower than that in the US patients, but slightly higher than that of Japanese hemodialysis patients [5, 16]. The study by Park et al. [7] may be of clinical importance because the results suggest the possible direct effects of ferritin independent of inflammation. However, their study does not answer the question whether or not systemic inflammation affects the association between serum ferritin and mortality risk.

Although we do not know precise mechanisms for the observed difference in the serum ferritin–mortality association between those having different degrees of inflammation, some explanations are possible. As discussed by Park et al. [7], ferritin appears to reflect some systemic conditions, other than inflammation, that could result in increased mortality and other adverse outcomes. Serum ferritin was reported to be increased in the presence of renal cell carcinoma [17] and other malignancies [18]. Also, increased tissue ferritin levels in the general [19] and in hemodialysis populations [20] are believed to indicate oxidative stress and tissue damage induced by iron released from ferritin. Furthermore, ferritin can act as a suppressor of immune system [21] which is involved in not only infectious disease but also cardiovascular disease. Therefore, even in the absence or at a very low grade of inflammation, an elevated level of serum ferritin could predict poor clinical outcomes independent of the markers of inflammation, as shown in the present study. On the other hand, it is known that there is considerable overlapping in the roles of inflammation, oxidative stress and PEW in prognosis of patients with CKD [14]. In addition, serum ferritin and CRP concentrations correlate with each other to a moderate extent [7]. Therefore, among subjects with signs of inflammation or at a higher grade of inflammation, it may be difficult to separate the roles of ferritin and inflammation in epidemiologic studies, as we report in this study.

In addition to the serum ferritin–mortality relationship, we examined the possible modification by the presence of inflammation of the association between serum ferritin and CVD hospitalization. Unexpectedly, we observed a linear and positive association between serum ferritin and CVD hospitalization in the total cohort as well as in any strata by CRP or serum albumin. These results indicate that the presence of inflammation does not modify the relationship between serum ferritin and CVD hospitalization. We have no clear explanation for the discrepancy between all-cause mortality and CVD hospitalization as endpoints. However, these observations raise a possibility that the effect modification by inflammation occurs in deaths not from CVD, but from infectious disease and cancer. Anyway, it is an important finding that higher serum ferritin predicted higher risk of hospitalization due to CVD in hemodialysis patients regardless of inflammatory status.

We noticed the death risk was higher in those with serum ferritin <50 ng/mL as compared with those with serum ferritin between 50 and 99.9 ng/mL in both strata of CRP <0.3 mg/dL and serum albumin ≥3.8 g/dL. On the other hand, such an increase in mortality risk in the lowest ferritin category was not found in strata of CRP ≥0.3 mg/dL or serum albumin <3.8 g/dL. These results indicate that both low and high serum ferritin levels are predictive of mortality risk in hemodialysis patients without signs of inflammation, and such relationship is hardly seen in the presence of inflammation. Although we have no clear explanations why serum ferritin <50 ng/mL was associated with higher mortality risk in hemodialysis patients without signs of inflammation, at least two explanations are possible. First, since such relationship was not found for CVD hospitalization, the higher risk of death in such subgroup of patients may have derived from non-CVD causes, such as gastrointestinal disease, causing chronic blood loss and iron deficiency. This speculation is supported by the cause of death in the four categories of serum ferritin stratified by CRP. Second, iron deficiency itself may be hazardous. In the general population, the harm of iron deficiency is reported even in those without anemia. Iron supplementation to non-anemic women decreased the degree of fatigue [22, 23] and sleep disturbances [23], both of which are known predictors of worse long-term outcomes in hemodialysis patients [24, 25]. To our knowledge, no previous cohort study reported the relationship of iron deficiency itself with mortality in the general population and other populations. Thus, this study is the first that suggests the potential harm of iron deficiency in the less inflamed subpopulation of hemodialysis patients.

The U-shaped association between serum ferritin and mortality in hemodialysis patients without signs of inflammation in this study appear somewhat different from the report by Kalantar-Zadeh et al. [2], showing that the mortality risk was lower in the lowest range of serum ferritin than in serum ferritin between 100 and 199 ng/mL. We speculate that the discrepancy between these studies can be explained by the different statistical models used in these studies. The US study [2] used a time-dependent model in which serum ferritin and the markers of inflammation and malnutrition were included as time-varying variables. In contrast, we used the traditional baseline model and the inflammation markers were handled as stratification factors based on our research question. It should be noted that baseline models give the associations in a long run, whereas time-dependent models indicate the associations (not always causality) in a short term [26]. We interpret the results by Kalantar-Zadeh et al. [2] to indicated that hemodialysis patients with a low ferritin (<50 ng/mL) are less likely to die in a short term as compared with those with serum ferritin of 100–199 ng/mL regardless of the presence of inflammation. In contrast, our results can be interpreted to indicate that hemodialysis patients with serum ferritin <50 ng/mL without inflammation have an increased risk of death in a long term, whereas such association between low ferritin and mortality was not seen in the presence of inflammation. We performed additional analyses with time-dependent models in which serum ferritin, ESA dose, and IV iron dose were included as time-varying variables which were updated quarterly. In such models, we could not detect the increased risk of death in the lowest ferritin category (<50 ng/mL) in the stratum without inflammation, but the mortality risk of the highest category of serum ferritin was significantly higher than that of the reference ferritin category in stratum with signs of inflammation (data not shown). These results with time-dependent models are similar to the report by Kalantar-Zadeh et al. [2], supporting the above interpretation of the results. Thus, our finding is novel and cannot be made without stratification of the cohort.

It is unknown whether or not a change in serum ferritin due to management of anemia brings on increased risk of the adverse events. Increased serum ferritin concentrations may result from higher dosing of IV iron or lower dosing of ESA [3]. In the US, serum ferritin level is reported to be higher than that before the implementation of the new bundle payment system [3], but no significant increase was found in mortality rate in the US hemodialysis population [27]. The Japanese Society for Dialysis Therapy (JSDT) has published the revised 2015 version of JSDT guideline [28]. In patients having hemoglobin lower than the target range, the new JSDT guideline: (1) proposes iron replacement prior to treatment with ESA in patients not treated with ESA or iron, if serum ferritin is <50 ng/mL; (2) suggests iron replacement therapy in patients treated with ESA, if serum ferritin is <100 ng/mL and transferrin saturation is <20 %, and (3) suggests iron replacement therapy when no condition is identified that could reduce iron utilization rate, if serum ferritin is <100 ng/mL or transferrin saturation is <20 %. As compared with the 2008 version of the JSDT guideline [12], which recommended iron replacement be initiated only in patients with serum ferritin ≤100 ng/mL and transferrin saturation ≤20 %, these revised statements may result in more cases being treated with iron and possibly higher serum ferritin levels than before. On the other hand, the new JSDT guideline does not recommend iron replacement therapy by which serum ferritin goes up to 300 ng/mL or higher. We do not know what happens in Japanese hemodialysis patients, who are less inflamed than those in the US, if the practice pattern is changed and serum ferritin level becomes higher. We need careful follow-up of the nationwide changes in serum ferritin concentration, mortality rate, and their relationship in patients with and without signs of inflammation.

This study has some limitations. First, because of the observational design, the results of this study do not necessarily imply causality. Second, we used serum ferritin concentrations by a single measurement at baseline. Also, we did not take the possible changes during the follow-up period into consideration. Therefore, our results could have underestimated or overestimated the true association. Third, because of the limited number (85) of deaths in the stratum with serum albumin ≥3.8 g/dL, the 95 %CI was large.

In conclusion, we revealed different patterns of association of serum ferritin and mortality in hemodialysis patients with versus without systemic inflammation. Combined with the known international difference in serum CRP and other inflammation markers, this finding may explain the apparent discrepancy in the association between serum ferritin concentration and mortality among countries. Further studies are needed to determine whether the results of this observational study can be translated into the management of anemia for hemodialysis patients.
